# Crossing the Vascular Wall: Common and Unique Mechanisms Exploited by Different Leukocyte Subsets during Extravasation

**DOI:** 10.1155/2015/946509

**Published:** 2015-10-19

**Authors:** Michael Schnoor, Pilar Alcaide, Mathieu-Benoit Voisin, Jaap D. van Buul

**Affiliations:** ^1^Department of Molecular Biomedicine, Center for Investigation and Advanced Studies of the National Polytechnic Institute (Cinvestav), 07360 Mexico City, DF, Mexico; ^2^Molecular Cardiology Research Institute, Tufts Medical Center and Tufts University School of Medicine, Boston, MA 02111, USA; ^3^Centre for Microvascular Research, William Harvey Research Institute, Barts & The London SMD, Queen Mary University of London, London EC1M 6BQ, UK; ^4^Department of Molecular Cell Biology, Sanquin Research and Landsteiner Laboratory, Academic Medical Center, University of Amsterdam, 1066 CX Amsterdam, Netherlands

## Abstract

Leukocyte extravasation is one of the essential and first steps during the initiation of inflammation. Therefore, a better understanding of the key molecules that regulate this process may help to develop novel therapeutics for treatment of inflammation-based diseases such as atherosclerosis or rheumatoid arthritis. The endothelial adhesion molecules ICAM-1 and VCAM-1 are known as the central mediators of leukocyte adhesion to and transmigration across the endothelium. Engagement of these molecules by their leukocyte integrin receptors initiates the activation of several signaling pathways within both leukocytes and endothelium. Several of such events have been described to occur during transendothelial migration of all leukocyte subsets, whereas other mechanisms are known only for a single leukocyte subset. Here, we summarize current knowledge on regulatory mechanisms of leukocyte extravasation from a leukocyte and endothelial point of view, respectively. Specifically, we will focus on highlighting common and unique mechanisms that specific leukocyte subsets exploit to succeed in crossing endothelial monolayers.

## 1. Introduction

**Figure 1:**
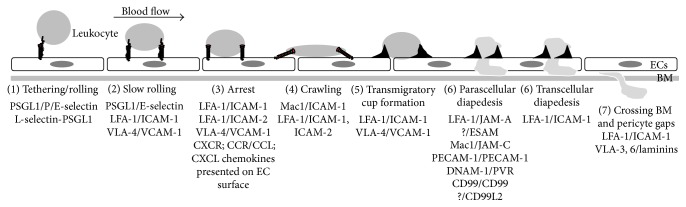
General scheme of the leukocyte extravasation cascade. The different steps of leukocyte interactions with endothelial cells during adhesion and transmigration are depicted. The known adhesion receptor interactions are listed for each step with the leukocyte receptor being named first. Unknown ligands are represented by question marks. During rolling, secondary rolling of leukocytes on already adherent leukocytes can occur that involve interactions of leukocyte L-selectin with leukocyte PSGL1 (not depicted). All receptors are connected to the actin cytoskeleton via actin-binding proteins to facilitate the extensive actin remodeling required for the morphological changes and movement of both cell types involved (not depicted). For details, see text.

The inflammatory response is critical for fighting infections and wound healing and is thus indispensable for survival [1, 2]. However, continuously active immune responses precede chronic inflammatory disorders and other pathologies. Thus, the immune response to injury and infection needs to be tightly controlled. In order to specifically interfere with excessive leukocyte transendothelial migration (TEM), a detailed understanding of the regulation of this multistep process is required. Butcher and Springer proposed in timeless reviews a multistep model for the process of TEM [3, 4]. Currently, this proposed model is still valid; however, over time some additional steps have been added to the sequence of events during TEM [2]. The inflammatory response starts with secretion of proinflammatory mediators such as histamine or cytokines that induce the opening of endothelial cell (EC) contacts in postcapillary venules to allow for passage of blood molecules, for example, complement factors. Inflammation also involves surface expression of endothelial adhesion molecules, actin remodeling, and activation of leukocyte integrins that enable leukocyte adhesion onto the endothelium within the vascular wall and subsequent diapedesis [5–8]. The sequence of adhesive interactions of leukocytes with EC is termed leukocyte extravasation cascade and involves a series of adhesive interactions that allow first tethering, rolling, and slow rolling, followed by firm adhesion, crawling, and transmigratory cup formation on the apical endothelial surface (Figure 1). Next is the actual TEM of leukocytes (also termed diapedesis) that can occur by crossing either EC contacts (paracellular) or the body of EC (transcellular). Both ways exist and it is known that the strength of endothelial junctions controls route preference [9] but the exact underlying mechanisms remain elusive. After crossing the endothelium, leukocytes also have to cross the pericyte layer and the basement membrane (BM) to reach the inflamed tissue and contribute to clearance of infection and wound healing [10]. Different types of leukocytes are being recruited to sites of inflammation including neutrophils, monocytes, and lymphocytes. In response to an inflammatory stimulus, neutrophils are generally among the first leukocytes to exit the blood stream, and, after degranulation, they contribute to a second wave of transmigration by mainly monocytes [11]. The reverse case has also been observed, in which the presence of monocytes and monocyte-derived neutrophil chemoattractants were required for neutrophil recruitment to sites of sterile inflammation [12]. Recruitment of all of these leukocyte subsets is compulsory for a proper immune response since all fulfill different functions once recruited to the inflamed tissue [13]. All these leukocyte types follow the sequential steps of the extravasation cascade in general, but differences in responsiveness to certain chemokines and in expression/activation of adhesion molecules to mediate interactions with EC have been described [8, 14]. Several mechanisms during the leukocyte extravasation cascade such as certain receptor-ligand interactions or signaling pathways have been confirmed as being exploited by all leukocyte subsets. However, other mechanisms have so far only been described for a single type of leukocyte. Whether these mechanisms are indeed unique for a given leukocyte subset or whether it has just not been studied yet in other leukocyte subsets is an important question to be answered in the future. A plethora of reviews have been published that summarize several aspects of leukocyte recruitment but in a generalized form that speaks only of “leukocytes.” In this review, we summarize current knowledge on common and unique mechanisms that different leukocyte types such as neutrophils, monocytes, and lymphocytes exploit during extravasation (Table 1). This includes signals induced within each leukocyte subset as well as differential signals that each leukocyte subset induces in EC to facilitate transmigration.

## 2. Mechanisms Exploited by Neutrophils to Achieve Extravasation

Representing ~40–60% of circulating leukocytes in the blood of humans, released at a rate of ~1-2 × 10^11^ cells per day into the blood stream and with a lifespan of only 1–5 days [15], neutrophils are among the first leukocytes to be recruited at sites of inflammation and/or injury. Migration of these unique leukocytes through blood vessel walls is a tightly regulated process for which some of the molecular interactions with the different components of the vessel wall (e.g., endothelium, pericyte sheath, and the venular BM) have been relatively well described in the literature [5, 14, 16]. There are now 5 major steps considered for the recruitment of neutrophils, namely, (1) capture and rolling along the luminal side of the endothelium, (2) firm adhesion and crawling toward the site of TEM, (3) TEM (and its variations), (4) subendothelial crawling along the pericytes processes, and (5) exit into the extravascular space through pericyte gaps and specific regions within the vascular BM. For many decades, it was assumed that chemokines and other soluble chemoattractants were responsible for the specificity of recruitment of leukocyte subsets due to a unique repertoire of G-protein coupled receptors present on their surface [17–19]. However, recent compelling* in vivo* evidences have challenged this idea and demonstrated a role for many adhesion molecules present on the EC surface specifically instructing the neutrophil to extravasate [20–22].

### 2.1. Capture and Rolling

Free flowing neutrophils are isolated from the endothelium by a dense, 0.5 to 5 *μ*m thick, network of negatively charged proteoglycans, glycosaminoglycans, and glycoproteins called the EC glycocalyx [23]. This structure acts as a formidable barrier for emigrating leukocytes and exceeds the dimensions of cellular adhesion molecules involved in neutrophil recruitment. Alterations of the EC glycocalyx are therefore a prerequisite for the first steps of neutrophil extravasation [24, 25]. Expression of positively charged molecules such as MPO on the surface of neutrophils [26] as well as shedding of the EC glycocalyx by heparinase [27], release of neutrophil-derived reactive oxygen species (ROS) [28], and matrix metalloproteinases (MMP) [29] contribute to facilitation of neutrophil-EC contacts. Once the EC glycocalyx is removed, neutrophils can reach the endothelial surface via a specific class of 3 closely related glycoproteins called the selectins and their glycoconjugate ligands (P-selectin glycoprotein ligand-1 (PSGL-1), CD44, and E-selectin ligand-1 (ESL-1)) [21, 30, 31]. L-selectin is constitutively expressed on the surface of neutrophils, whereas P- and E-selectins are more specific to EC. P-selectin is constitutively stored in distinct EC granules called Weibel-Palade bodies that are rapidly mobilized to the EC surface where P-selectin gets homogeneously distributed on the cells. By contrast, E-selectin is synthetized* de novo* during activation and concentrated mainly at EC junctions. Interestingly, a new study from Zuchtriegel and colleagues [22] demonstrated that neutrophils mainly use P-/L-selectin and PSGL-1/CD44 but not E-selectin to tether and roll along the endothelium* in vivo*. When blocked, these interactions affected not only the flux of rolling neutrophils but also their subsequent firm adhesion, crawling, and TEM. By contrast, inflammatory monocytes additionally need to interact with E-selectin for proper transmigration across the vessel wall, thus highlighting a new singular difference in the molecular interactions between different subsets of leukocytes and EC during this first stage of transmigration.

Despite the weak and transient nature of the molecular interactions between selectins and their ligands, neutrophils roll even under high shear stress within blood vessels. Sundd and colleagues have recently made some interesting observations on how these neutrophils maintain contact with EC during rolling [32]. During the initial contacts with EC through selectins/selectin ligands, the structure of the neutrophil cell membrane is modified by reorganization of both cytoskeleton and surface adhesion molecules leading to the formation of an extended protrusion called sling [33]. This structure is formed from a membrane tether at the back of the rolling neutrophil like an anchor before it is wrapped around the rolling leukocyte and swings to the front of the cell to recontact the EC. Such slings contained heterogeneous patches of PSGL-1 conferring intermittent adhesive structures to the EC surface but are also rich in the *β*2-integrin lymphocyte function-associated antigen-1 (LFA-1). Furthermore, the binding of PSGL-1 to P-/L-selectin during the rolling step leads to conformational changes in the neutrophil *β*2-integrin LFA-1 through outside-in signalling [34–36]. This response allows for binding of LFA-1 to its ligands on EC supporting slow rolling and eventually transition to firm adhesion of the neutrophil [37].

### 2.2. Firm Adhesion and Crawling

Strengthening of neutrophils-EC interactions occurs mainly through the binding of the leukocyte *β*2-integrins LFA-1 and macrophage-antigen-1 (Mac1) to their cognate receptor intercellular adhesion molecule-1 (ICAM-1) expressed on activated EC [38]. These interactions enable the neutrophils to firmly adhere to the surface of the endothelium. In parallel to *β*2-integrins/ICAM-1 adhesive complexes, the neutrophil *β*1-integrin very late antigen-4 (VLA-4) and its EC binding partner vascular cell adhesion molecule-1 (VCAM-1) can contribute to the arrest of leukocytes in specific inflammatory conditions in humans [39]. This strengthened adhesion is completed by the sensing of chemotactic molecules such as chemokines (e.g., CXCL1/2), lipid mediators (e.g., LTB4, PAF), and complement proteins (e.g., C5a) by G-protein-coupled receptors (GPCRs) on the surface of the neutrophils that further signal through the cytoskeleton to induce full activation of the integrins and firm adhesion [37]. Following this firm adhesion, neutrophils crawl perpendicular to or even against the flow of the bloodstream, toward chemotactic [40] (e.g., chemokines) or haptotactic (e.g., ICAM-2) gradients. The mechanism of this luminal crawling is strictly ICAM-1/Mac1-dependent [41, 42] as blockade of these two molecules* in vivo* resulted in neutrophils failing to both crawl and migrate through EC junctions without affecting neutrophil adhesion. It has been suggested that the transition between LFA-1-dependent firm adhesion and Mac1-dependent crawling of neutrophils occurs via inside-out signalling through LFA-1 and the activation of the guanine exchange factor Vav-1 [43] that consequently activates Mac1 [44]. Recently, another member of the CAM family, ICAM-2, has been shown to play a role in neutrophil crawling dynamics toward EC junctions prior to TEM [45]. In mice exhibiting genetic deletion of this molecule as well as in WT animals treated with a blocking antibody against ICAM-2, neutrophils exhibited an increase in crawling duration and reduced crawling speed, leading to neutrophils lingering longer along the luminal surface of EC and delaying their migration through endothelial junctions.

### 2.3. TEM and Its Variations

TEM is the most rapid response of the migration cascade of neutrophils, lasting 5–10 min depending on the inflammatory scenario. Several molecular interactions between neutrophils and EC have been described for this step in the literature [5, 14, 16]. The penetration of EC by neutrophils occurs via two routes: through EC-EC intercellular junctions (i.e., paracellular migration) or through the body of the EC (i.e., transcellular migration). Recent* in vivo* evidence showed the predominance of the paracellular route (90% of transmigration events) over the transcellular migration [46]. Genetically modified mice in which the adherens junctions and more particular the VE-cadherin-catenin/VE-PTP complex are stabilized showed that the blood vessel wall became impermeable to macromolecules and neutrophil infiltration [47, 48]. By contrast, mice deficient for the actin-binding protein cortactin showed reduced clustering of ICAM-1 around adherent neutrophils due to defective activation of the GTPase RhoG in EC leading to strongly reduced adhesion and transmigration [49, 50]. Numerous adhesion molecules enriched at EC-EC junctions such as PECAM-1, JAM family members, ICAM-2, CD99, ESAM, and CD99L2 are involved in the process of neutrophil TEM. These molecules are also detected in subcellular structures called the lateral border recycling compartment (LBRC) that play a key role in neutrophil TEM [51, 52]. In basal conditions, these adhesion molecules contribute to the maintenance of EC junctions; however, during inflammation they engage with their counter-receptors on neutrophils (e.g., *β*2-integrins LFA1 and Mac1 and through homophilic interactions of PECAM-1, JAM-A, or CD99 that are also expressed on leukocytes) to allow for crossing of the junctions in a sequential manner [16, 53, 54]. The binding of adhesion molecules between neutrophils and EC can also mediate polarization signals in the neutrophils allowing them to correctly migrate from the luminal to abluminal sides of the EC. This is particularly true for JAM-A [55] and JAM-C [56]. Two recent publications demonstrated* in vivo* the presence of abnormal transendothelial migratory events [46, 57] characterized by the neutrophil partially migrating through the junction with oscillatory movements in the junction (i.e., hesitant migration) or even returning back to the circulation in an abluminal-to-luminal direction (reverse migration) following ischemia-reperfusion injury or leukotriene B4- (LTB4-) induced inflammation. This abnormal transmigration can represent up to 20% of total TEM events. This response could be reproduced or even enhanced in other inflammatory conditions in the absence or by blockade of JAM-C [46]. Abnormal transmigration indeed involves the removal of JAM-C from the junction via cleavage by neutrophil elastase [57] following its translocation from azurophilic granules to the surface of the leukocyte in a complex with the integrin Mac1 upon direct stimulation of the neutrophil by LTB4 [58]. Genetic deletion or pharmacological inhibition of NE could significantly restore the presence of JAM-C at junctions and reduce the abnormal transmigration events. On the other hand, exogenous injection of NE in inflammatory models not known for exhibiting abnormal TEM of neutrophils increased the number of these events. This specific abnormal TEM response was associated with the presence of soluble JAM-C in the serum and an increase in secondary organ damage, two key features regularly observed in patients with trauma or ischemia-reperfusion injury.

### 2.4. Abluminal Crawling

Earlier observations of migration events showed that, after TEM, the vessel wall was thickening and neutrophils could only be detected in the tissue more than 20–40 min after TEM had occurred. For many decades, nothing was known about what was happening to the neutrophil during this period of time. Once migrated through the EC, the abluminal neutrophil faces the second cellular component, that is, the pericytes, and its tight matrix, the venular basement membrane (BM), in which they are embedded [59]. Many of the studies of transmigration events have neglected these two components of blood vessel walls due to the difficulty to reproduce the complete structure* in vitro* or to visualize it* in vivo*. However, recent developments of new advanced microscopy techniques and the generation of genetically fluorescent animals have shed new lights on the role of pericytes in the recruitment of neutrophils* in vivo*. Pericytes express adhesion molecules and chemokines such as ICAM-1, VCAM-1, and CXCL1 upon inflammation both* in vivo* and* in vitro* [60–63]. This response was correlated with the observations that, after TEM, neutrophils were found crawling along pericyte processes away from their site of TEM in an ICAM-1/Mac1- (and to a lesser extent LFA-1) dependent manner before fully breaching the venular wall [63]. Blocking those molecular interactions with blocking antibodies could suppress both neutrophil abluminal motility and their entry into the interstitial space.

### 2.5. Exit from the Vessel Wall

Following abluminal crawling, neutrophils exit the vessel wall through specific enlarged gaps between adjacent pericytes. The role of pericyte gap enlargement is still unclear, but, interestingly, less than 10% of the gaps were used by migrating neutrophils and most of the time hot spots of transmigration could be observed where more than 2-3 neutrophils exited via the same pericyte gaps. It has been suggested that potential enrichment in adhesion molecules and chemokines around specific pericyte gaps [63] as well as the release/generation of chemoattractants by the leading neutrophils from their granules [11] and/or from the cleavage of BM proteins into chemotactic fragments [64] could pave the way for subsequent neutrophils.

The venular BM (generated by both the EC and the pericytes) is the final interactive matrix (but also barrier) for emigrating neutrophils. This structure is composed of tight networks of matrix proteins such as collagen type IV and laminins [65]. Interestingly, blocking the interactions between leukocyte integrins VLA-3 and VLA-6 (receptors for collagen and laminin, resp.) and the venular BM using blocking antibodies could inhibit the migration of neutrophils through this layer [66–68]. Another unique characteristic of neutrophil interaction with the venular BM is the discovery of low expression regions (LERs) within the BM that are preferred sites for neutrophil migration [10, 59]. These sites contain low quantities of matrix proteins, are associated with gaps between adjacent pericytes, and are being used and enlarged during neutrophil, but not monocyte, migration [69]. In fact, it will take another 10 to 20 minutes more for the neutrophils to migrate through pericyte gaps and LERs as observed* in vivo*, with many oscillatory movements by the neutrophils [63]. However, the duration of LER/pericyte gap penetration and oscillatory movements are reduced for the subsequent neutrophils following the same hot spot of migration. Though the mechanism of the remodelling of such permissive sites in the BM of venular walls is not fully understood, it has been suggested that proteolytic cleavage by neutrophil enzymes [10, 59] and/or reversible disassembly of collagen fibres [65] could be involved in this process, thus allowing the neutrophil to finally access the interstitial space.

## 3. Mechanisms Exploited by Monocytes to Achieve TEM

### 3.1. Monocyte Populations

Monocytes are heterogeneous cells that circulate in the blood in distinguishable populations termed resident (or patrolling) and inflammatory monocytes according to the expression profile of certain chemokine receptors and adhesion molecules [70–72]. While resident monocytes are associated with immune surveillance and wound healing, inflammatory monocytes are connected to induction and maintenance of inflammatory immune responses [73]. On the other hand, monocytes give rise to dendritic cells and macrophages to promote inflammatory responses [74, 75]. Monocytes are actively being recruited from the bone marrow via the blood stream to inflamed tissues in a largely CC-chemokine receptor 2/CC-ligand-2-(CCR2/CCL2) dependent fashion. Very recently, an elegant intravital imaging study reported a phenotypic conversion of monocyte subsets at sites of sterile liver injury [76]. First, inflammatory monocytes were rapidly recruited and stayed around the injured site for about 48 h before a conversion to a resident monocyte phenotype and entry into the injured area occurred to induce wound healing. This previously unrecognized monocyte plasticity highlights the importance of monocytes for resolution of inflammations. Furthermore, a targeted silencing approach using nanoparticles containing CCR2-specific siRNA has been described in mice that prevented accumulation of inflammatory monocytes at sites of inflammation and ameliorated various pathological conditions in which inflammatory monocytes have been implicated [77]. This is a promising approach to specifically target inflammatory monocytes without affecting other immune cells during inflammation; however, it remains to be proven whether such an approach is applicable to humans.

### 3.2. Rolling and Slow Rolling

TEM of monocytes occurs according to the paradigm of the leukocyte extravasation cascade as described above [78]. In addition to the well-established role of PSGL-1 in all leukocyte rolling, monocyte rolling during recruitment to lymphoid tissues also depended on L-selectin and CD44 [79]. In the infected skin, proper monocyte rolling and subsequent recruitment depended on monocyte PSGL-1 interaction with endothelial E- and P-selectin, whereas monocyte L-selectin interacted with endothelial peripheral node addressin (PNAd) [80]. L-selectin shedding is required at later stages of transmigration to ensure a regulated and polarized conclusion of transmigration [81]. On EC expressing high amounts of VCAM-1, for example, in atherosclerotic lesions, monocyte rolling and transition to slow rolling and arrest strongly depended on the *β*1-integrin VLA-4 [82, 83]. However, on ECM-bound platelets, monocyte rolling rather depended on the *β*2-integrin Mac1 and P-selectin [84].

### 3.3. Firm Adhesion

Once slowed down, leukocytes recognize chemokines presented on the endothelium that lead to GPCR-mediated inside-out signaling, full integrin activation, and the subsequent arrest of leukocytes. The endothelial Duffy antigen receptor for chemokines (DARC) has recently been reported to transport CCL2 across the endothelium to the apical side where it contributed to proper monocyte activation and recruitment [85]. In monocytes, full VLA-4 activation after GPCR stimulation depended on a signaling axis including phospholipase C (PLC), inositol-1,4,5-triphosphate (IP3), Ca2+-flux, and calmodulin but not on PI3K [86], which is in contrast to VLA-4 activation in neutrophils. Although the adhesion cascade has been best studied in neutrophils (reviewed in [8]), a common denominator regulating the transition from rolling via slow rolling to arrest in all leukocytes is the activation of PLC. However, depending on the stimulus, the primary integrin inducing firm adhesion/arrest varies among leukocyte subsets. In the case of monocytes, it seems to be VLA-4. Recently, growth differentiation factor-15 (GDF-15) has been identified as an endogenous inhibitor of VLA-4 activation that prevented monocyte binding to VCAM-1 and could thus serve as a local inhibitor of inflammation [87]. VCAM-1 expression and thus monocyte adhesion are increased by the endothelial receptor protein tyrosine kinase EphA2 [88, 89]. Other examples of endogenous monocyte integrin inhibitory molecules are the endothelial matrix protein developmental endothelial locus-1 (Del-1) and endothelial CD47 interacting with monocyte signal regulatory protein-*α* (SIRP-*α*) [90, 91]. Thus, monocyte-endothelial interactions seem to be regulated at different levels and most likely other regulation mechanisms will be unraveled in the near future.

### 3.4. Crawling

After firm adhesion, leukocytes spread and crawl on the endothelial surface to find a suitable spot for transmigration. This process has first been observed with monocytes and was called locomotion [92]. Such directional movement of monocytes preceding transmigration could be blocked by antibodies against LFA-1 or Mac1 strongly suggesting dependence on *β*2-integrins. In the case of patrolling monocytes, antibodies against LFA-1 but not Mac1 detached crawling monocytes, an effect that was also observed in mice lacking CX3CR1 [72]. In unstimulated cremaster venules, more monocytes (compared to neutrophils) adhered and crawled for longer distances in an LFA-1-dependent manner. Once stimulated by tumor necrosis factor-*α* (TNF-*α*), monocytes reduced their crawling distance that now became Mac1-dependent and more neutrophils crawled in a strictly Mac-1-dependent fashion [93]. In this study, Mac1 blockage was more efficient in reducing both monocyte and neutrophil extravasation compared to LFA-1 blockage. While crawling of monocytes can be both LFA-1-dependent and Mac1-dependent, neutrophil crawling is strictly Mac1-dependent [42], thus marking a striking difference between these leukocyte subsets during extravasation.

### 3.5. Docking Structures or Transmigratory Cups

During inflammatory leukocyte recruitment, the activated endothelium supports neutrophils by forming clusters around adhering leukocytes that are enriched in LFA-1/ICAM-1 and VLA-4/VCAM-1. These structures first appear as ring-like structures that surround adherent leukocytes and later engulf leukocytes as docking structures or transmigratory cups that are enriched in actin and various adaptor molecules such as cortactin, ezrin, radixin, moesin- (ERM-) proteins, and filamin, and signaling molecules such as RhoG and Rac1 [49, 50, 94–100]. Another endothelial adhesion receptor found in docking structures is activated leukocyte cell adhesion molecule-1 (ALCAM-1) that supports monocyte recruitment into the central nervous system (CNS) [101]. Docking structures have been observed* in vitro* with all leukocytes.* In vivo*, similar structures, so-called domes, have been described for neutrophils [102]. For more details, see the endothelial part below.

### 3.6. Diapedesis

In order to cross the endothelial monolayer between intercellular junctions, the VE-cadherin/catenin complex needs to be disassembled as it constitutes a physical barrier for transmigrating leukocytes [47]. Real-time imaging of transmigration* in vitro* using VE-cadherin-GFP overexpressing HUVEC revealed that monocytes as well as neutrophils used preexisting and* de novo*-formed VE-cadherin gaps to achieve paracellular transmigration [103]. Interestingly, initial transmigration of monocytes causes downregulation of VE-cadherin and upregulation of PECAM-1 that facilitates subsequent monocyte transmigration [104]. Other molecules of the interendothelial junctions can actually be exploited as counter-receptors by transmigrating leukocytes to facilitate transmigration. For example, monocyte LFA-1 can bind to JAM-A and JAM-A deficiency greatly reduces transmigration of monocytes [105, 106]. In the brain, blocking JAM-A interactions with LFA-1 reduced transmigration of monocytes and neutrophils and ameliorated the overall neurological damage after ischemia/reperfusion injury [107]. Expression of JAM-like protein (JAM-L) is upregulated on monocytes during inflammation and binds to the endothelial receptor coxsackie and adenovirus receptor (CAR), an interaction that is regulated in* cis* by VLA-4 [108, 109]. Other adhesion molecules within EC contacts that serve as counter-receptor during inflammatory monocyte recruitment are PECAM-1, CD155, and CD99. PECAM-1 and CD99 interact homophilically, whereas endothelial CD155 interacts with CD226 on monocytes. Blockade of all these molecules that are part of the lateral border recycling compartment (LBRC, [52]) by antibodies drastically reduced monocyte TEM without affecting rolling, adhesion, and crawling [110–112]. Interestingly, these molecules act in a sequential manner with PECAM-1 engagement occurring first followed by CD155 and then CD99 [112]. Moreover, occludin in the tight junction controls monocyte transmigration across the blood-brain barrier in response to methamphetamine in an actin-related protein-2/3- (Arp2/3-) dependent fashion [113]. Inhibiting Arp2/3 prevented methamphetamine-induced occludin-internalization and monocyte TEM.

### 3.7. Postdiapedesis Events

In order to cross the BM and the pericyte layer, both monocytes and neutrophils exploit areas of pericyte gaps and low matrix protein expression within the BM [10, 69]. However, while neutrophils were capable of enlarging these low matrix protein expression regions, monocytes showed increased deformability and rather squeezed through them [69]. NG2^+^-pericytes also guide and bind transmigrated monocytes and neutrophils by chemokine secretion and ICAM-1 expression, respectively [114]. A common mechanism for all leukocytes after crossing the EC layer is the elongation of uropods and a delayed detachment of the leukocyte from the basal endothelial surface [68]. This process depended on LFA-1 interaction with abluminal ICAM-1 that maintained the connection of the uropod with the endothelium and, on the other hand, on VLA-3 that mediated movement through the BM. Signaling required for tail retraction after diapedesis involves RhoA [115]. Inhibiting RhoA in monocytes renders the cells unable to complete diapedesis and leads to *β*2-integrin accumulation in the unretracted tail. Vice versa, RhoA activation in EC is also required for efficient monocyte transmigration [116] which involves ROCK-mediated myosin light chain phosphorylation and disruption of VE-cadherin-dependent EC contacts [117]. Monocytes that fully transmigrated express the surface receptor discoidin domain receptor-1*α* (DDR1*α*) that can bind collagen and seems to facilitate monocyte migration within collagen-rich extracellular matrices (ECM) in inflamed tissues [118]. Interestingly, monocytes can undergo reverse transmigration when JAM-C-mediated adhesion is disrupted by antibodies* in vivo* [119].

## 4. Mechanisms Exploited by Lymphocytes to Achieve TEM

T and B lymphocytes (T and B cells) are adaptive immune cells capable of recognizing and distinguishing antigens leading to functional specificity and memory. T and B cells arise from the bone marrow and populate the peripheral lymphoid organs (spleen and lymph nodes), where they complete maturation and become activated in response to specific antigens presented by antigen presenting cells, including dendritic cells (DCs). Trafficking to the lymph nodes occurs via lymphocyte L-selectin-dependent adhesion to its ligands on high endothelial venules (HEV), glycan-bearing cell adhesion molecule-1 (GlyCAM-1), and CD34, followed by integrin-dependent arrest via LFA-1 and VLA-4. The affinity of these integrins is rapidly increased by the chemokines CCL19 and CCL21 that are mainly produced in lymphoid tissues by HEV. These chemokines then bind to CCR7 expressed in both naïve T cells and B cells. Upon antigen presentation, naïve T and B cells become activated and lose L-selectin expression [120–122]. Activated lymphocytes can either become effector cells or memory cells. Effector B cells produce antibodies that contribute to the elimination of extracellular microbes. Effector T cells can be separated into CD8^+^ cytotoxic T cells, which kill virus-infected cells, and subsets of CD4^+^ T cells including T helper (Th) cells that help other immune cells carry out their functions, and T regulatory (Treg) cells that suppress the functions of effector T lymphocytes. T and B cell subsets are further divided into well-defined subtypes that play different roles in health and disease [123]. In this section, we summarize the different mechanisms exploited by T and B cells to achieve transendothelial migration. Extravasation of T cell subsets has been studied in more detail* in vitro* and* in vivo*. B cells also infiltrate sites of inflammation in several diseases [124–126] but it is assumed that B cells largely exploit mechanisms similar to those of T cells. Current studies focusing on B cell extravasation are discussed at the end of this section.

### 4.1. Mechanisms of T Cell Extravasation

T lymphocytes (T cells) are specialized leukocytes that have the ability of recognizing antigens and participate in the adaptive immune response. The process of T cell recruitment to sites of infection or injury is fundamental in the immune response to exogenous antigens, with implications for responses to autoantigens that trigger chronic tissue inflammation when immune tolerance is disrupted in autoimmunity [127]. Within the T cell recruitment cascade, T cell adhesion is perhaps the best-understood process under flow conditions, with several* in vitro* studies involving human CD3^+^ T cells and T cell-like cell lines characterizing the molecular signals regulating chemokine-induced integrin activation [128]. These* in vitro* studies using human T cells have also demonstrated that the behavior of T cells differs from other leukocytes once contact with the activated vascular endothelium is established. T cells randomly adhere to the apical side of the endothelium and locomote on the surface for some time. This is thought to be required to allow for enough time to induce the chemokine-chemokine receptor crosstalk necessary for optimal T cell integrin activation and subsequent TEM [129]. This contact time also allows the T cell receptor (TCR) to recognize potential antigens being presented by vascular EC, a recently explored pathway that can also lead to TEM [130]. Recent studies on different T cell subsets expanded the classic paradigm of T cell recruitment by identifying novel critical molecules and pathways leading to T cell extravasation* in vivo*. These involve common but also T cell-specific molecules that participate in the different steps of the T cell recruitment cascade as summarized below.

#### 4.1.1. Rolling

Rolling of T cell subsets has mostly been characterized using CD4^+^-T cells, with the understanding that many findings may also hold true for CD8^+^-T cells [131, 132]. As in other leukocytes, T cell rolling occurs under shear conditions by interactions of endothelial selectins with highly glycosylated T cell-expressed selectin ligands. In contrast to other leukocytes such as neutrophils and monocytes, which constitutively express all the glycosyltransferases required for functional selectin ligand biosynthesis, these are normally inducible and regulated in T cells [21]. Therefore, the ability of T cells to bind endothelial selectins is acquired in response to signals that assure the proper glycosylation of selectin ligands. These signals include specific cytokines, and thus T helper type 1 (Th1) cells and T helper type 2 (Th2) cells, which require different cytokines for differentiation and survival [133, 134], differ in the initial rolling step of the T cell recruitment cascade due to the differential expression of active selectin ligands. Th1 cells are major players in immune responses to intracellular microbes and in tissue damage associated with autoimmunity and chronic infections. They express high levels of glycosyltransferases in response to the Th1 cytokine IL-12 and thus have highly glycosylated selectin ligands for rolling on the activated endothelium. Th2 cells contribute to fight against helminth infections and autoimmune atopic diseases but have a rather low extravasation potential compared to Th1 cells [135–137]. The more recently discovered Th17 cells participate in the immune response to extracellular bacteria and fungi and, similarly to Th1 cells, play a role in organ-specific autoimmunity and chronic inflammation [138]. Interestingly, both Th1 and Th17 cells share selectin ligands such as PSGL-1 to roll on the vascular endothelium via P-selectin [139]. More recent data indicate that these subsets, in contrast to the Th2 or naïve cells, can also use the T cell immunoglobulin and mucin domain 1 protein (TIM-1) as a P-selectin ligand to mediate T cell trafficking during inflammation and autoimmunity [140]. Similarities and differences in rolling mechanisms are observed in E-selectin-ligand/E-selectin mediated interactions among T cell subsets, with Th17 cells being more dependent on E-selectin-mediated interactions than Th1 cells [139]. Besides PSGL-1 which functions similarly in Th1 and Th17 cells as a ligand for both E-selectin and P-selectin, other E-selectin ligands have been identified in Th1 cells that are functional only in cooperation with PSGL-1, and these include CD44 [141] and CD43 [142, 143]. Further research will determine whether these can also function in Th17 cells in a subset specific manner.

#### 4.1.2. Adhesion

The emerging novel roles for different T cell subsets in acute and chronic inflammatory processes and the differential expression of chemokine receptors in different T cell subsets have recently been recognized as being critical for integrin-mediated adhesion in response to specific chemokine-chemokine receptor signaling [144]. The integrin associated protein (CD47) has been shown to regulate adhesive functions of the *β*2-integrins LFA-1 and VLA-4 in T cells* in vitro*. This was proven to be a critical mechanism regulating T cell adhesion to the cremaster microvasculature* in vivo* in studies involving competitive recruitment of CD47 wild type and CD47-deficient Th1 cells [145]. The integrin coactivator Kindlin-3 has also recently been shown to reinforce T cell integrin activation and adhesion. Interestingly, this mechanism is specific for T cell adhesion but did not play a role in T cell diapedesis [146]. Studies using mouse Th1 and Th17 cells generated* in vitro* have also demonstrated that these cells express a different repertoire of chemokine receptors. Depending on the chemokine ligand exposed, these two subsets adhered to immobilized ICAM-1 only in the presence of SDF1*α* and CCL20, respectively, under shear flow conditions* in vitro*. Other studies using human T cells and HUVEC also demonstrated that CCL20 mediates Th17 adhesion to EC [147]. These studies nicely correlate with* in vivo* studies showing a role for CCR6, the receptor of CCL20, in specific CCR6^+^-Th17 cell recruitment to the gut [148], to the central nervous system [149], and to the skin [139]. These adhesion mechanisms are therefore chemokine/chemokine receptor-specific. They may also be tissue specific, as Th17 cells in liver endothelium utilize vascular adhesion protein-1 (VAP-1) and the chemokine receptor CXCR3 in addition to CCR6 to mediate adhesion via *β*1 and *β*2 integrins [150].

#### 4.1.3. Diapedesis

T cell TEM follows rolling and adhesion and ultimately leads to T cell infiltration into inflamed sites. Compared to other leukocytes such as neutrophils, the percentage of T cells that undergo TEM in* in vitro* assays under flow conditions is much smaller, and it normally requires additional chemokines such as SDF1*α* in order to facilitate T cell arrest and TEM. Similarities with other leukocytes include ICAM-1-mediated signaling upon T cell adhesion, and VE-cadherin gaps at the site of junctional transmigration [151–153]. Recent work has demonstrated that T cells can trigger the dissociation of the endothelial receptor phosphatase VE-PTP from VE-cadherin as a mechanism leading to VE-cadherin phosphorylation and gap formation to facilitate transmigration [154]. Endothelial CD47 can also promote VE-cadherin phosphorylation and participate in T cell transmigration* in vitro*. Interestingly, CD47 expressed on T cells is also required for T cell TEM* in vitro* and for T cell recruitment at sites of dermal skin inflammation* in vivo* [155].* In vitro*, VAP-1 can mediate TEM of T cells across liver EC [156]. More recent studies have demonstrated that VAP-1 together with the common lymphatic endothelial and vascular endothelial receptor (CLEVER-1) and ICAM-1 can specifically regulate TEM of Treg cells [157]. VAP-1, CLEVER-1, and ICAM-1 are highly expressed at sites of leukocyte recruitment to the inflamed liver suggesting that they also regulate T cell transmigration into the liver* in vivo*. From all these studies it is clear that chemokines presented by the endothelium are critical for integrin-dependent adhesion and TEM of effector and memory T cells* in vitro*. This has significant implications* in vivo*, where chemokine gradients are present in the context of infection or injury leading to T cell transmigration. A novel alternative mechanism described for T cell transmigration is the ability of effector T cells to access not only extracellularly deposited chemokines, but also intraendothelial chemokines such as CCL2 stored in vesicles inside the EC to achieve transmigration [158].

As different T cell subsets were identified as principal players in chronic inflammation, a role of the vascular endothelium has been considered as critical for the migratory patterns acquired by antigen-experienced effector T cells that migrate to sites of chronic inflammation. Whether these pathways are T cell subset specific or organ/vascular bed/disease-specific remains to be investigated [164]. Given that EC express major histocompatibility complex molecules (MHC) I and II and therefore can function as antigen presenting cells for both CD4^+^- and CD8^+^-T cells, it is recently being recognized that, at the time of contact and antigen presentation, EC can imprint restricted, specific trafficking molecules in T cells. These are thought to be acquired in the organ where the T cells were generated [165], or at the site of infection or inflammation, where these antigenic signals are thought to contribute to the recruitment of these T cell subsets [166, 167]. Thus, the classic paradigm of chemokine-induced T cell arrest and TEM is now being challenged with alternative ways that T cells use to achieve TEM in different inflammatory contexts.* In vitro*, both effector and memory CD4^+^- and CD8^+^-T cells dynamically probe the endothelium by extending actin-rich invadosome/podosome like protrusions (ILPs) [168], thought to actively participate in TEM by distorting the actin filaments and breaching the endothelial barrier [9].* In vivo* mouse studies have shown that antigen specific CD4^+^-effector T cells use cognate antigen driven signals presented by MHC-II for entry into pancreatic islets in autoimmune diabetes [169, 170]. Apical presentation of cognate antigenic peptides by MHC-I and perivascular dendritic cells are thought to increase integrin adhesiveness and TEM of CD8^+^-T cells in vascular beds deficient in adhesive and chemotactic activities such as the pancreatic islets in diabetes [167] and vascularized transplants [171].* In vitro* studies under flow conditions using human effector and memory CD4^+^-T cells have contributed to gain further insight into the mechanisms taking place in TEM mediated by antigen presentation and TCR signals versus classic chemokine-induced TEM not involving antigenic signals. These studies have demonstrated that T cells can rapidly transmigrate in response to both chemokines and TCR-activating antigenic signals, but these two mechanisms differ in some of the molecular pathways regulating TEM: TCR stimulated TEM was highly dependent on fractalkine (CX3CL1), PECAM-1, CD99, nectin-2, poliovirus receptor (CD155), and ICAM-1, whereas chemokine-stimulated TEM involved ICAM-1 and JAM-A but not any of the other molecules [172]. Furthermore, both of these TEM pathways triggered the activation of the protein ZAP-70 in the transmigrating T cell but differ in the signaling downstream of ZAP-70. Vav-1, Rac-1, and myosin 2A activation occurred only in the T cells that have been in contact with vascular EC in an antigen-TCR dependent way [173]. Phenotypically, this signaling resulted in different T cell cytoskeleton reorganization during transmigration, with the T cell microtubule organizing center (MTOC) being organized in the contact region between the T cell and the EC. Dynein-driven transport of granzyme-containing granules to the contact region between the T cell and the EC was identified as the mechanism regulating the T cell cytoskeleton reorganization during TEM [174]. Thus, these specific molecular signals observed in TCR-driven T cell transmigration closely resemble immune synapse formation and seem to be a novel process that T cells utilize to achieve successful TEM.

Taken together, T cells are uniquely specialized to respond to antigens, proliferate, and differentiate into subsets that acquire migratory phenotypes that allow them to traffic to sites of inflammation previously accessed by neutrophils and monocytes. T cells share some of these recruitment mechanisms with other leukocytes and trigger similar signals on the vascular endothelium to achieve TEM. The specialized T cell response to different antigens and the cytokine milieu results in distinct expression of active selectin ligands and a different repertoire of chemokine receptors involved in rolling and arrest on the vascular endothelium. Once adhered to the endothelium, they can use classic TEM routes and novel antigen-dependent routes. Understanding the mechanisms that regulate the recruitment of effector T cells in different inflammatory settings will shed new light on potential ways these pathways can be exploited for immunotherapeutic purposes.

### 4.2. Mechanisms of B Cell Extravasation

As mentioned above, B cells utilize in general the same basic mechanisms as naïve T cells to home to secondary lymphoid organs. How activated B cell subsets migrate into specific tissues during inflammation has not been explored in such detail as for T cell subsets, for which each step of the recruitment cascade has been analyzed* in vitro* and* in vivo*. However, some studies have identified some differences in inflammatory B cell extravasation as compared to T cells that will be discussed here. Many mature B cells, named plasma cells, migrate from the lymph nodes to the bone marrow, where they secrete IgG antibodies for long periods of time that are distributed through the body via the blood stream. This B cell subset expresses VLA-4 and CXCR4, which bind to VCAM-1 and CXCL12, respectively, expressed in bone marrow sinusoidal endothelial cells. In contrast, mature B cells that produce IgA antibodies, express *α*4*β*7, CCR9, and CCR10 which bind to MadCAM-1, CCL25, and CCL28, respectively, expressed in mucosal endothelial cells to migrate to mucosal tissues such as the gut [175, 176]. These molecules are also thought to mediate IgG and IgA-producing B cell recruitment to sites of chronic inflammation in the synovium [124], the brain [125], and the vessel wall [126]. Moreover, CXCL12 was able to stimulate diapedesis of human B cells across human brain microvascular endothelial cells under flow conditions, and this was blocked by CXCR4 function blocking antibodies [159]. Recently, ADAM28, which is highly expressed in B cells but not in T cells, has been shown to bind to VLA-4 and to increase VLA-4-dependent adhesion of the murine B lymphoma cell line L1-2 to VCAM-1 and subsequent transendothelial migration suggesting that this metalloprotease affects the efficiency of B cell extravasation [177]. Interactions of ephrin-A4 with its endothelial receptor EphA2 have also been shown to regulate normal as well as leukemic B cell transendothelial migration [178]. B cells are also present within chronically inflamed liver tissue. Using* in vitro* flow adhesion assays and hepatic sinusoidal endothelial cells, human blood-derived B cells were captured via VCAM-1 without requiring a previous rolling phase and remained static before achieving transendothelial migration mediated by ICAM-1, VAP-1, and the chemokine receptors CXCR3 and CXCR4. This mechanism represents a prominent difference in B cell extravasation, since T cells displayed vigorous crawling before transmigration in the same system [160]. Others have observed so-called “intraendothelial canalicular” structures that are especially exploited by B cells to cross the endothelium during homing and inflammation [179]. Whether these structures can also be observed during the extravasation of other leukocyte types needs to be analyzed in the future.

## 5. The Dynamics of Leukocyte Diapedesis from an Endothelial Point of View

Under quiescent conditions, the endothelium expresses low levels of adhesion molecules, allowing limited immune surveillance. However, upon encounter of an infection or tissue damage, surveilling monocytes and neutrophils are triggered to release inflammatory cytokines, such as TNF-*α* and IL1*β*. The importance of this has been recently underscored by the group of Dr. Nourshargh [180]. They showed in an elegant* in vivo* model that neutrophils locally secreted TNF-*α* which immediately acted on the endothelium and thereby assisted other neutrophils in transmigration. For longer periods of inflammation, EC respond to inflammatory mediators by massive upregulation of adhesion receptors, such as P-selectin, E-selectin, ICAM-1, and VCAM-1, whereas ICAM-2 was found to be decreased [181, 182]. These adhesion molecules attract circulating immune cells to adhere to and transmigrate through the endothelial monolayer (Figure 1). Moreover, inflammatory cytokines induce presentation of chemoattractants on the EC surface, such as CXCL4/5 and IL-8 [14]. During rolling, G-protein-coupled receptors on the leukocytes encounter such chemokines presented by the EC and signal to induce a conformational change of the leukocyte integrins LFA-1 (*α*_*L*_ *β*_2_) and VLA-4 (*α*_4_ *β*_1_) into a high affinity state enabling these integrins to interact with their endothelial ligands ICAM-1 and VCAM-1, respectively [13, 183]. As a consequence, firm adhesion, crawling, and finally diapedesis occur via interendothelial junctions or through the EC body [184]. Although these processes are believed to occur for any type of leukocyte that crosses the endothelium, some endothelial signals are specifically induced by certain leukocyte subsets that we will highlight in this chapter.

### 5.1. Adhesion Molecule Upregulation

The inflammatory cytokine TNF-*α* stimulates EC by binding to its receptor TNFR1 (CD120a) [185, 186]. This binding induces the association of the cytoplasmic adaptor protein TRADD (TNFR1-Associated Death Domain protein) to the intracellular domain of TNFR1. Subsequently, TRADD binds to downstream effectors such as the serine/threonine kinase RIP1 (Receptor Interacting Protein 1), as well as the E3-ubiquitin ligase TRAF2 (TNFR-associated Factor 2). This association in turn triggers a kinase signaling cascade leading to the activation of the mitogen-activated protein kinases (MAPKs) p38, JNK, and ERK [187]. These kinases are able to activate transcription factors, such as activator protein-1 (AP-1). In addition, TRAF2 and RIP1 induce the activation of the transcription factor NF-*κ*B. Under quiescent conditions, NF-*κ*B is retained in the cytosol by inhibitor of *κ*B (I*κ*B*α*). Upon activation of the I*κ*B*α* kinase (IKK) complex by TRAF2 and RIP1, I*κ*B*α* is phosphorylated, which leads to its degradation and the subsequent nuclear translocation of NF-*κ*B [188].

The promoters of the adhesion molecules E-selectin, VCAM-1, and ICAM-1 contain several NF-*κ*B-binding sites, and NF-*κ*B has been shown to be the primary regulator of TNF-*α*-induced adhesion molecule expression in EC [189–194]. Although the promoters of E-selectin, VCAM-1, and ICAM-1 also contain AP-1-binding motifs, these sites have varying contributions to TNF-*α*-induced upregulation of these adhesion molecules [192, 193, 195, 196]. In addition, other transcription factors such as Interferon Regulatory Factor-1 (IRF-1), Specificity protein 1 (Sp1), and GATA are also known to become activated via poorly characterized signaling pathways and contribute to TNF-*α*-induced adhesion molecule upregulation in EC [191, 197–199].

### 5.2. Signaling by CAMs

The integrin expression patterns differ per leukocyte type. For example, neutrophils primarily express Mac1 and LFA-1 and hardly any VLA-4, whereas monocytes and also T-lymphocytes and dendritic cells express all three integrins, albeit at different levels [200]. This already indicates that different leukocyte types through their integrin repertoire can cluster different ligands on the endothelium resulting in distinctive intracellular signals in the endothelium that differ per leukocyte type. For example, Th17-lymphocytes showed increased adhesion to E-selectin compared to Th1-lymphocytes, most likely because of a better integrin activation on these cells through the CCL20-CCR6 axis [139].

**Figure 2:**
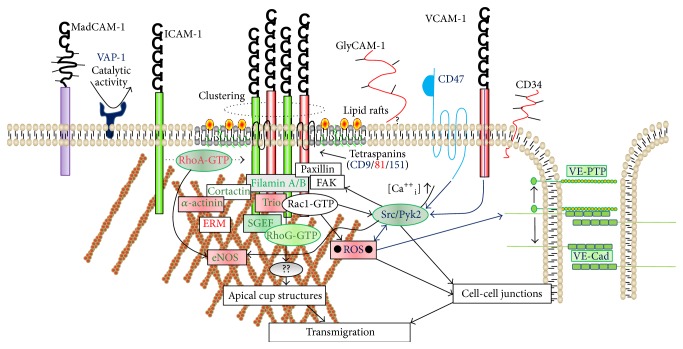
Endothelial signaling pathways induced upon clustering of ICAM-1 and VCAM-1 leading to the formation of endothelial F-actin-rich apical cup structures and the dissociation of endothelial adherens junctions. Endothelial signals that are induced by specific leukocyte types are color-coded: neutrophils in green, monocytes in red, T cells in blue, and B cells in purple. In case signaling proteins are identified by studies using different leukocyte types, the background color of the protein name is adapted to the leukocyte type used. In case specific signaling is studied in the absence of leukocytes the color is black. Short stripes indicate glycosylation. Question mark indicates that GlyCAM-1, as a soluble protein, may reassociate to the endothelial membrane.

ICAM-1 and VCAM-1 are members of the immunoglobulin (Ig) superfamily of adhesion molecules, whose extracellular domains are characterized by the presence of five and six Ig-like domains, respectively. Compared to their ectodomains, ICAM-1 and VCAM-1 have relatively small carboxyl- (C-) terminal intracellular domains of only 28 and 19 amino acids, respectively. Although the C-terminal domains do not contain any apparent signaling motifs, the intracellular domain of ICAM-1 has been shown to be required for efficient leukocyte TEM [201, 202]. Moreover, ICAM-1 engagement by LFA-1/Mac1 has been linked to F-actin reorganization and to the initiation of signaling events within EC [203]. Several studies have shown that leukocyte adhesion and clustering of ICAM-1 induced an increase in intracellular Ca^2+^ levels [204, 205] leading to activation of the tyrosine kinase Src by protein kinase C (PKC). In turn, Src induced tyrosine phosphorylation of focal adhesion proteins such as paxillin, cortactin, and FAK [204, 206, 207]. ICAM-1 clustering led to the activation of the small RhoGTPase RhoA which stimulated the formation of F-actin stress fibers and increased endothelial monolayer permeability [204, 208–210] (Figure 2). Moreover, RhoA activity was also demonstrated to be required for efficient ICAM-1 recruitment around adherent monocytes [210] suggesting an upstream role for RhoA within the ICAM-1-induced signaling cascade. Recently, it was shown that ICAM-1 clustering induced tyrosine phosphorylation of VE-cadherin in a Src- and Pyk2-dependent manner, which coincided with increased endothelial permeability [203, 211, 212]. Martinelli and colleagues showed that ICAM-1 clustering induced the phosphorylation of eNOS on S1177 and this was regulated by Src kinase, as well as RhoA, calcium, CaMKK, and AMP kinase, but not PI3 kinase. They additionally showed that this pathway controlled the phosphorylation of VE-cadherin and lymphocyte trafficking [203]. In contrast to ICAM-1, only a few studies have reported signaling events induced upon engagement and clustering of VCAM-1. The leukocyte integrin VLA-4, expressed on monocytes and lymphocytes, showed strong binding preference for VCAM-1 [213]. Clustering of VCAM-1 was shown to promote activation of Rac1 leading to the production of reactive oxygen species (ROS) [214–216]. VCAM-1-dependent ROS production was demonstrated to regulate the activation of matrix metalloproteases, which may contribute to the local breakdown of the endothelial adherens junctions [217]. In addition, VCAM-1 clustering was shown to regulate lymphocyte TEM by activation of the kinase PKC*α* and the tyrosine phosphatase PTP1B in a ROS-dependent manner [218, 219].

In addition to the classical adhesion molecules on the endothelium (e.g., ICAM-1/2 and VCAM-1), several other molecules are known to play an important role in leukocyte traffic. Several of them belong to ectoenzymes, which are cell surface molecules having catalytically active sites outside the cell. For example, the adhesion molecule Vascular Adhesion Molecule-1 (VAP-1) with amine oxidase enzymatic activity was discovered to be present at the endothelial surface and controls the traffic of lymphocytes [220–222], monocytes [161], and neutrophils [162, 163]. However, if these ectoenzymes transmit intracellular signals into the endothelium that remodel the actin cytoskeleton during leukocyte TEM is not known.

The transmembrane protein CD47 is also an important mediator of leukocyte trafficking [145, 155]. CD47 is expressed on many if not all leukocyte types as well as EC and interacts with SIRP*γ* that is expressed on lymphocytes [223]. The same group showed that CD47 can phosphorylate VE-cadherin and in this way mediate lymphocyte TEM, again in a Src- and Pyk2-dependent manner [155]. Interestingly, cross-linking of CD47 with antibodies led to formation of stress fibers, similar to what has been observed when cross-linking ICAM-1 [209, 210, 224]. Clearly, changes in the endothelial actin cytoskeleton induced by leukocyte binding control efficient leukocyte TEM.

Tetraspanins form microdomains in the plasma membrane and are involved in intercellular adhesion and migration. For lymphocyte and monocyte TEM, it has been reported that the tetraspanins CD9, CD81, and CD151 distribute to the contact site with transmigrating leukocytes and associate laterally with both ICAM-1 and VCAM-1 [225, 226]. They control the adhesive capacity of the adhesion molecules and thereby control leukocyte binding strength to the endothelium. In addition, Barreiro and coworkers found that tetraspanins can form so-called endothelial adhesive platforms (EAPs) to which leukocytes can bind [95]. These platforms can function as signaling hubs in the plasma membrane and may include lipid rafts as well. Interestingly, ICAM-1 and VCAM-1 can both be present in these platforms, independent of the presence of its receptor.

A summary of the above-described signaling pathways downstream of clustered ICAM-1 and VCAM-1 is shown in Figure 2, where we have color-coded the endothelial proteins that are activated by specific leukocyte subsets.

### 5.3. CAM Linkage to the F-Actin Cytoskeleton

To support proper adhesion under physiological flow conditions, ICAM-1 and VCAM-1 need to be intracellularly anchored to the cytoskeleton. In the past two decades, several actin adapter proteins have been reported to interact with the intracellular domains of VCAM-1 and ICAM-1. These adapter proteins link these molecules to the F-actin cytoskeleton (Figure 2). The adapter proteins ezrin and moesin from the ERM-family were found to interact with VCAM-1 in a direct manner. Moreover, they colocalized with VCAM-1 around adherent lymphoblasts [94]. Their ability to bind both phospholipids and F-actin allows ERM proteins to organize adhesion molecules into specialized membrane domains [227]. In addition to VCAM-1, ERM proteins were also reported to interact with ICAM-1 in a PIP_2_-dependent manner and colocalized with ICAM-1 in microvilli-like structures [94, 210, 228, 229]. However, unlike the binding to VCAM-1, the interaction of ezrin and moesin with ICAM-1 was reported to be indirect [230]. In addition to ERM proteins, the F-actin bundling proteins *α*-actinin-1 and -4 were also demonstrated to interact with the ICAM-1 C-terminus through a cluster of ICAM-1-C-terminal positively charged amino acids [231, 232]. Interestingly, this same cluster of amino acids was shown to mediate the interaction of ICAM-1 with ezrin [229] suggesting that *α*-actinin and ERM proteins may compete for binding to ICAM-1. This also indicates the existence of different ICAM-1/actin complexes upon leukocyte-mediated clustering (Figure 2).

The cortical actin-binding protein cortactin was initially shown to become tyrosine phosphorylated upon ICAM-1 clustering [206] and this tyrosine phosphorylation was required for efficient neutrophil TEM [100]. Cortactin is thought to stabilize branched actin networks through interaction with the Arp2/3 complex [233]. It also associated with ICAM-1 upon clustering [234] and was required for ICAM-1 and F-actin recruitment to ring-like structures around adherent leukocytes [99]. Recently, it was shown that cortactin is also required for ICAM-1 clustering around adherent neutrophils and for efficient neutrophil extravasation* in vivo*, thus highlighting the physiological relevance of the ICAM-1-cortactin interaction [49].

Finally, Kanters and colleagues showed that the F-actin cross-linker protein filamin B interacts with the ICAM-1 C-terminus in a direct manner [235]. Similar to cortactin, filamin B was required for ICAM-1 recruitment to a ring around adherent neutrophils and for neutrophil TEM under physiological flow conditions. In a more recent publication, it was shown that also filamin A interacts with the intracellular tail of ICAM-1 [236]. It has therefore been proposed that these adapter proteins connect ICAM-1 to downstream signaling partners [237, 238]. Silencing of filamin B expression impaired ICAM-1 clustering and leukocyte TEM under physiological flow conditions and since filamin A was still present in filamin B-silenced EC, this suggests that the filamins are not functionally redundant.

Indeed, although filamins A and B share 70% amino acid sequence identity, different effects on ICAM-1 function were observed when either filamin A or filamin B was silenced. Using fluorescence recovery after photobleaching (FRAP) technology, it became clear that silencing of filamin B expression increased the immobile fraction of ICAM-1 in the plasma membrane [235]. In contrast, in EC lacking filamin A, the immobile fraction of ICAM-1 was reduced [239]. Additionally, the clustering-induced ICAM-1-actin association was impaired in filamin A-silenced EC, but not in filamin B-silenced EC (personal observation, JDvB). The effects of filamin A deficiency on ICAM-1 function are similar to those of inhibition of F-actin polymerization. Moreover, deletion of the intracellular domain of ICAM-1 decreased the immobile fraction of ICAM-1 [239]. In addition, filamin A, but not filamin B, also mediated the interaction of ICAM-1 with the lipid raft marker and main constituent of caveolae, caveolin-1 [235]. Since ICAM-1 is recruited to caveolae and caveolin-1 during transcellular lymphocyte TEM [240], filamin A may have a specific role in regulating the transcellular pathway of diapedesis. These findings therefore reveal important roles for the different filamins in controlling ICAM-1 dynamics by regulating the connection with the F-actin cytoskeleton and specific membrane domains.

Recently, it became clear that there is a hierarchy between these actin-binding proteins to bind to ICAM-1 upon clustering. Schaefer and colleagues showed that when ICAM-1 is clustered, *α*-actinin is the first protein to be recruited to ICAM-1, followed by cortactin and lastly filamin [241]. The recruitment of different adapter proteins to ICAM-1 may result in the composition of a different actin network. For example, *α*-actinin cross-links actin filaments into actin bundles whereas cortactin cross-links actin into a meshwork and filamin into a “gel-like” structure [233, 237]. The initiation of these different actin networks may generate forces that drive local protrusive activity, that is, docking structures, or create a surface for leukocytes to crawl on. In fact, they showed that the local stiffness of the EC was indeed dependent on *α*-actinin. Depleting *α*-actinin resulted in reduced ability of the neutrophils to spread and transmigrate [241]. The group of Dr. Carman showed recently that the cytoskeletal morphology and as a consequence the local EC stiffness of different vascular beds determined the preferred route for T-lymphocytes to cross the endothelium [9]. In particular, high barrier function was associated with transcellular migration, whereas artificial opening of the junctions resulted in more paracellular migration. They previously showed that T-lymphocytes use invadopodia-like protrusions to probe the endothelial surface, possibly to initiate transcellular migration [242]. It is tempting to speculate that the rate of clustering of adhesion molecules like ICAM-1 or VCAM-1 determines the stiffness of the underlying endothelial surface and that this may be the trigger for, at least, T-lymphocytes to cross. Whether other leukocyte types, for example, neutrophils or monocytes, use the same mechanism to probe the surface is not known.

A recent study by the Woodfin group showed that, next to ICAM-1 and VCAM-1, ICAM-2 plays an important role in immune cell traffic* in vivo* as described above [45]. Whether clustering of ICAM-2 recruits actin adapter proteins and induces similar signals is not clear [202]. However, the role of ICAM-2 seems to be more restricted to certain organs. For example, endothelial ICAM-2 is required for the migration of T-cells across the blood-brain barrier [243, 244].

### 5.4. Endothelial Docking Structure Formation

Using confocal microscopy, Barreiro and coworkers showed that both ICAM-1 and VCAM-1 were recruited to actin-rich membrane protrusions that surround adherent T-lymphoblasts in cup-like structures that were termed endothelial docking structures [94]. Two subsequent studies by Carman and coworkers demonstrated that the formation of these structures was dependent on F-actin polymerization and correlated strongly with transmigrating leukocytes [96, 97]. They suggested that these structures may function to facilitate and guide leukocyte TEM by forming a cup-like traction structure that is aligned parallel to the direction of transmigration. Interestingly, they showed that the transmigratory cups were essentially equal between monocytes, neutrophils, and lymphocytes. Thus, the cups did not discriminate between the leukocyte types suggesting that these endothelial cups represent a more global mechanism for leukocyte extravasation. In contrast to what Barreiro and coworkers proposed, that is, that the docking structures are required for leukocyte adhesion, Carman and colleagues showed that the cups were highly associated with leukocytes that transmigrated. Disruption of the cups did not alter the capacity of leukocytes to adhere to the endothelium, even under flow conditions. Interestingly, the formation of the cups depended on the intracellular tail of ICAM-1 [98]. In line with the notion that cups are not involved in adhesion, several reports have shown that the intracellular tail of ICAM-1 is needed for proper diapedesis but not for firm adhesion [201, 202, 229]. In addition to the* in vitro* observations, numerous studies have also described the formation of endothelial docking structures* in vivo* [102, 245–249]. Thus, although definite proof is still lacking, the formation of docking structures shows a strong correlation with the diapedesis step.

The initial formation of endothelial docking structures is dependent on the activity of the small GTPase RhoG [238]. RhoG colocalized with ICAM-1 upon ICAM-1 clustering and got activated. Moreover, depletion of SGEF, a GEF for RhoG, or RhoG significantly reduced the formation of docking structures and neutrophil TEM. Using a murine model to study the formation of atherosclerosis, it became clear that SGEF is most likely involved in the recruitment of monocytes to the site of injury since SGEF-deficient animals showed a significant reduction of plaques compared to control animals [250]. This work highlights the importance of docking structures in the development of inflammation-based diseases such as atherosclerosis.

Interestingly, Doulet and colleagues showed that the signaling molecules that are normally responsible for the induction of the apical cup structures can be used by bacteria (e.g.,* Neisseria meningitides*) to enter EC [251]. These bacteria titrated the actin adapter protein ezrin and moesin away from sites where leukocytes interacted on the endothelium and thereby prevented the formation of cup structures and leukocyte diapedesis. This study shows the potential clinical relevance of cup structures in leukocyte TEM during inflammation. How these pathogens manage to cross the vessel wall and enter host cells can tell us a lot on the basic principles of the signaling mechanisms during leukocyte TEM.

## 6. Conclusions

The initial multistep paradigm of leukocyte extravasation largely describes adhesion and diapedesis from a leukocyte point of view and regarded the endothelium merely as just a passive substrate for leukocyte adhesion. However, it is now well appreciated that the endothelium is also an active participant in this process. Clustering of adhesion molecules, such as ICAM-1 and VCAM-1, has been demonstrated to induce signaling leading to significant changes in EC morphology allowing for leukocyte passage. In addition, endothelial cup structures are formed that may capture and guide leukocytes to transmigrate across the endothelium. Thus, the essential role of endothelial adhesion receptors and actin-binding proteins in mediating leukocyte TEM makes them promising candidates for a targeted regulation of leukocyte extravasation. On the other hand, several mechanisms in leukocytes have been identified that activate, for example, integrins for proper interactions with EC and actin dynamics causing the required morphological changes during TEM. Several of such mechanisms have been identified in all leukocyte subsets while others seem to be specific for a given subset. However, whether they are really specific or have just not yet been investigated in other subsets remains to be seen for most of the described mechanisms. It is important to keep in mind that all leukocyte types, besides their potential for tissue destruction, fulfill beneficial functions during many pathophysiological conditions so that pharmacological targeting of leukocyte recruitment will most likely always cause beneficial and detrimental effects. Thus, a lot of work remains to be done until we can fully appreciate whether there are truly unique mechanisms exploited by different leukocyte subsets during TEM that could be targeted pharmacologically in certain pathological conditions that would benefit from interference with the recruitment of only one given leukocyte type without affecting others.

## Figures and Tables

**Table 1:** Overview of some mechanisms that regulate extravasation of leukocyte subtypes in the order of events during the leukocyte extravasation cascade.

TEM step	Regulatory proteins	Cell	Function	Reference
Tethering/rolling/slow rolling	L-selectin, PSGL-1	EC/monos	L-selectin interacts with PNAd and PSGL-1 with P- and E-selectin to mediate proper rolling	[80]
	P-selectin, Mac1	EC/monos	Rolling and adhesion on ECM-bound platelets under flow	[84]
	P-selectin, PSGL-1, and CD44	EC/monos	Mediate rolling during monocyte recruitment to lymphoid tissues during inflammation	[79]
	CD44	Neutrophils/T cell	CD44 interacts with E-selectin in cooperation with PSGL-1 to mediate rolling	[141]
	TIM-1	T cell	TIM-1 interacts with PSGL-1 to mediate rolling	[140]
	CD43	T cell	CD43 interacts with E-selectin in cooperation with PSGL-1 to mediate rolling	[142, 143]
	P-selectin, PSGL1/PSGL-1 CD44, and L-selectin	ECs/neutrophils	Mediate rolling during recruitment of neutrophils in cremasteric postcapillary venules	[21, 22]
	PSGL-1, LFA-1/P-selectin, and ICAM-2	Neutrophil/ECs	Mediate sling formation and slow rolling	[32, 37]

Arrest/adhesion	VLA-4	Monos	PLC-, Ca-, and calmodulin-dependent arrest in response to chemokines	[86]
	VLA-4, GDF-15	Monos	GDF-15 reduces VLA-4 activation and monocyte adhesion	[87]
	LFA-1/ICAM-1	Neutrophils	Mediate neutrophil arrest	[42]
	EphA2	EC	Reduction of VCAM-1 expression and monocyte adhesion	[89]
	DARC	EC	CCL2 transport to the apical EC surface to induce monocyte activation and recruitment	[85]
	SIRP*α*/CD47	Monos/EC	Negatively regulates *β*2-integrin-mediated monocyte adhesion and transmigration	[91]
	CD47	T cell	Mediates integrin-dependent arrest on VCAM-1 and ICAM-1 and T cell recruitment *in vivo*	[145]
	Kindlin 3	T cell	Reinforces T cell adhesion	[146]
	CXCR4	Monos/B cell	CXCL12-dependent adhesion and diapedesis	[159]
	VCAM-1	B cell	VCAM-mediated arrest without rolling	[160]

Crawling	LFA-1, Mac1	Monos	Locomotion in search for the nearest suitable junction to start diapedesis	[92]
	LFA-1, Mac1	Monos	Crawling in unstimulated cremaster venules LFA-1-dependent that becomes Mac1-dependent after TNF-*α*-stimulation	[93]
	LFA-1; CCL3/CXC3CL1	Monos	Patrolling of resident monocytes and recruitment into noninflamed tissues	[72]
	Mac1/ICAM-1	Neutrophils	Control the luminal crawling of neutrophils on endothelial ICAM-1	[42]
	Mac1/ICAM-2	Neutrophils	Control the directionality and speed of crawling of neutrophils on endothelium	[45]

Cup formation	LFA-1/ICAM-1 VLA-4/VCAM-1 ALCAM-1	All	Clustering of these receptor-ligand pairs around adhering leukocytes causes GTPase activation, actin adaptor molecule recruitment, actin remodeling, and protrusion formation to engulf and support the adherent leukocyte	[49, 50, 94–100]

TEM	JAM-A, JAM-L, JAM-C, PECAM-1, DNAM-1, CD155, and CD99	All	Serve as counterreceptors for leukocyte-EC interactions during the passage through interendothelial cell contacts	[105, 108–112]
	Mac1, NE/JAM-C	Neutrophils/ECs	Control the directionality of neutrophil transendothelial migration. Cleavage of JAM-C induces aberrant transendothelial migration	[57]
	VAP-1	T cell	Together with ICAM-1 and CLEVER-1 specifically regulates T cell TEM	[156, 157]
		Monos	Support CX_3_CL1-dependent monocyte transmigration across hepatic sinusoidal EC	[161]
		Neutrophils	Blocking enzymatic activity of VAP-1 reduces neutrophil diapedesis and accumulation in lungs	[162, 163]
	Occludin	EC	Methamphetamine-induced Arp2/3 activation induces occludin internalization and monocyte transmigration	[113]
	JAM-A	Monos Neutrophils	Blocking JAM-A interaction with LFA-1 reduces recruitment of monocytes and neutrophils into the brain after ischemia/reperfusion injury	[107]

After TEM	CXCL1/ICAM-1 Mac1/LFA-1	Pericytes/neutrophils	Abluminal crawling along pericyte processes	[63]
	VLA-3 VLA-6/collagen, laminin	Neutrophils/venular BM	Control the migration of neutrophils through venular basement membrane and exit through LERs	[10]

Interstitial motility	LFA-1 VLA-3	All	Interaction with abluminal ICAM-1 enables uropod extension while VLA-3 mediates movement of the leading edge in the BM	[68]
	ICAM-1	Pericytes	NG2^+^-pericytes secrete chemokines and express ICAM-1 to attract/bind transmigrated leukocytes	[114]
	DDR1*α*	Monos	Expressed after transmigration *in vivo* to support migration within collagen-rich ECMs	[118]
	JAM-A	Neutrophils	Controls polarized interstitial migration	[55]
	RhoA	Monos	Active RhoA required for tail retraction to complete diapedesis	[115]
